# Characteristics of Sudden Unexpected Cancer Deaths Investigated by Medical Examiners in Tokyo, Japan (2009)

**DOI:** 10.2188/jea.JE20130087

**Published:** 2014-03-05

**Authors:** Hideto Suzuki, Takanobu Tanifuji, Nobuyuki Abe, Tatsushige Fukunaga

**Affiliations:** Tokyo Medical Examiner’s Office, Tokyo Metropolitan Government, Tokyo, Japan; 東京都監察医務院

**Keywords:** cancer, sudden death, medical examiner, mortality statistics, terminal care

## Abstract

**Background:**

Annually, about 400 cases of sudden unexpected death are attributed to cancer in Tokyo, Japan. These individuals may have been undiagnosed, or their medical conditions may not have been carefully evaluated before death. We examined medical consultations, cancer diagnoses, and economic status of all cancer deaths investigated by medical examiners in 2009.

**Methods:**

Among cases handled by the Tokyo Medical Examiner’s Office in 2009 (*N* = 12 493), records for all cases of cancer death (*n* = 400) were reviewed to determine the extent of medical care provided, diagnosis before death, and economic status of the decedent.

**Results:**

Most of the decedents (*n* = 232; 58%) had received a diagnosis of terminal/advanced cancer during a medical consultation. Most did not receive such medical consultations at home, despite their very weak physical condition. However, nearly one quarter of decedents (24%; 95/400) had not received a cancer diagnosis before death. The proportions of decedents who had been indigent, received no medical consulting, and had colon cancer were significantly higher among undiagnosed cases than among diagnosed cases. Indigent persons were the largest subgroup (*n* = 19; 43%) among those who had never received a medical consultation (*n* = 44). In addition, the proportion of those who had discontinued or received no medical consultation was higher among indigent persons than among non-indigent persons.

**Conclusions:**

The quality of medical services for cancer patients could be improved by educating general practitioners about terminal care, expanding efforts to monitor and diagnose cancer, especially among indigent patients, and increasing participation rates for colorectal cancer screening.

## Abstract (ja)

**（背景）:**

監察医制度が施行されている東京都23区では悪性腫瘍による異状死が毎年約400件発生しているが、当該事例の生前診断等の疫学的事項の詳細は明らかにされていない。

**（方法）:**

2009年に東京都監察医務院で取り扱った全異状死例から死因が悪性腫瘍である事例を抽出し（400例）、生前の医療機関受診状況・診断、経済状況につき調査した。

**（結果）:**

大部分の事例（232例、58%）は生前に終末期癌と診断されており、衰弱しているにも関わらずその多くは在宅医療を受けていなかった。一方、95事例（24%）は生前未診断であったが、生前診断群と比較すると生活困窮者、医療機関未受診者、大腸癌の割合が有意に高かった。生活困窮者は医療機関未受診者の43%を占め、医療機関受診状況に関して非生活困窮者と比較すると治療中断もしくは未受診の割合が有意に高かった。

**（結論）:**

悪性腫瘍に対する医療の質をさらに向上させるためには、開業医に対する終末期医療教育、生活困窮者に対する検診と医療の継続、大腸癌検診の受診率の向上が必要であると考えられる。

## INTRODUCTION

Cancer has been a leading cause of death in Japan since 1981, and in recent years more than 300 000 cancer deaths occur annually.^1^ The Ministry of Health, Labour and Welfare has developed a plan to address aspects of cancer management, including steps to improve prevention and early detection and expand cancer research.^1^^,^^2^

Most cancer cases are diagnosed, and patients receive various therapies, including surgery and palliative care, before death. However, in most cases cancer progresses, whereas other diseases, such as circulatory disease, can cause sudden death. According to statistics from the Tokyo Medical Examiner’s Office—where medical examiners scrutinize all cases of sudden unexpected death—about 400 such cases each year (about 3% of all deaths handled by that office) are determined to be due to cancer.^3^^–^^6^ This raises the important question of whether cancer diagnoses are missed before death in such cases or if such deaths are due to rare cancers, as was suggested in previous reports.^7^^–^^9^ However, this question has remained unanswered.

We examined medical consultations, cancer diagnoses, and economic status of all cancer deaths handled by medical examiners in Tokyo, Japan in the year 2009.

## METHODS

### Study sample

All sudden unexpected deaths (reported as sudden unexpected death from disease, non-disease-related causes, or unknown cause) are reported to the Tokyo Medical Examiner’s Office by the police in the special wards of the Tokyo metropolis, and medical examiners perform postmortem examinations to determine the manner and cause of death. Medical examiners perform autopsies when the cause of death cannot be determined by past medical history, course of illness, or situational and external investigations of the deceased. In the present study, we reviewed the records of cases handled by the Tokyo Medical Examiner’s Office during 2009. The study sample comprised cases for which the cause of death was cancer (*n* = 400). Records available for review included death certificates, medical examiner reports on postmortem examinations, reports of autopsies (if performed), and summaries of death scene investigations by police.

We examined age, sex, primary cancer site, and autopsy results (if performed) for each case. In addition, we compared the age distribution and primary cancer site of the cases with those for all cancer deaths in Japan, as described in the 2009 census report.^10^

### Medical consultation and lifetime cancer diagnosis

We examined whether decedents had consulted a doctor before death. If medical consultation was provided before death, we ascertained the diagnosis and determined if the patient had received home visits from the physician.

### Economic status

We ascertained economic status (eg, salaried or welfare recipients) of the patients in each case and calculated the proportion of people who were considered indigent, which was defined in this study as recipients of social welfare and homeless persons. A total of 75 decedents were classified as indigent: 67 had been welfare recipients and 8 had been homeless.

### Statistical analysis

Intergroup comparisons were performed using the χ^2^ test for independence or the *t* test. A *P* value of less than 0.05 was considered to indicate statistical significance.

## RESULTS

In total, 400 cases of sudden unexpected death due to cancer were included in this study. Of these, 165 (41%) required transportation by ambulance to a critical care center after being found unresponsive. After unsuccessful cardiopulmonary resuscitation, a physician in the critical care center reported cause of death to the police as death of unknown cause. The remaining 235 cases had postmortem changes when the ambulance arrived and were thus not transported to a critical care center; these cases were reported directly to the police.

Death due to cancer was most frequently reported in individuals aged 70 to 79 years (Figure). The proportions of deaths among individuals aged 60 to 69 years and those aged 70 to 79 years were slightly higher in the study sample as compared with all cancer deaths in Japan. In general, in both groups more than 80% of deaths were in people older than 60 years (Figure). Major primary cancer sites did not significantly differ between the study sample and all cancer deaths in Japan (Table 1).

**Figure.**
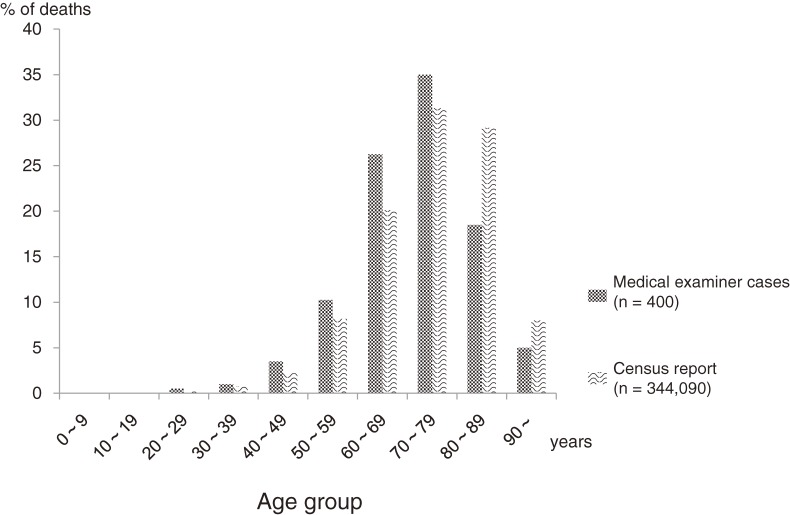
Age distributions of decedents and all cancer deaths in Japan (2009).

**Table 1.** Primary site of cancer

	Medical examiners’ cases	Census report
Lung	74 (19%)	67 583 (20%)
Stomach	54 (14%)	50 017 (15%)
Colon	44 (11%)	42 434 (12%)
Liver	36 (9%)	32 725 (10%)
Pancreas	31 (8%)	26 791 (8%)

Values in parentheses indicate percentages of the total (*n* = 400; *n* = 344 090).

More than three-quarters (76%; *n* = 305) of decedents had received a cancer diagnosis before death (Table 2), and 89% of those 305 people (*n* = 271) had received a diagnosis of advanced or late stage cancer before death. Cause of death was confirmed without autopsy in 95% (*n* = 290) of these cases (autopsy rate 5%, Table 2). Most individuals (217; 71%) had sought treatment at a large hospital; 19 (6%) had gone alone to medical clinics before death. Only 11 (4%) had received medical care at home, while 48 (16%) had elected to discontinue medical consultations (Table 3). Overall, 232 individuals (58% of all cases) had received a diagnosis of advanced or terminal stage cancer while alive and had not refused medical consultation.

**Table 2.** Characteristics of decedents

	Diagnosed (*n* = 305)	Undiagnosed (*n* = 95)	
Age (mean ± SD)	71 ± 12	70 ± 13	
Male	214 (70%)	63 (66%)	
Autopsied	15 (5%)	90 (95%)	**
Indigent person	49 (16%)	26 (27%)	*
Primary cancer site			
Lung	67 (22%)	7 (7%)	**
Stomach	37 (12%)	17 (18%)	
Colon	18 (6%)	26 (27%)	**
Liver	30 (10%)	6 (6%)	
Pancreas	23 (8%)	8 (8%)	
Others	130 (43%)	31 (33%)	

Values in parentheses indicate percentages of the total (*n* = 305; 95).

**P < 0.01, *P < 0.05.

**Table 3.** Medical consultation before death

	Diagnosed (*n* = 305)	Undiagnosed (*n* = 95)	
Medical consultation			
Hospital	217 (71%)	19 (20%)	**
Clinic	19 (6%)	11 (12%)	
Home visit by physician	11 (4%)	2 (2%)	
Unknown medical facility	10 (3%)	0	
Discontinued treatment	48 (16%)	17 (18%)	
No consultation	0	44 (46%)	**
Unknown	0	2 (2%)	*

Values in parentheses indicate percentages of the total (*n* = 305; *n* = 95).

**P < 0.01, *P < 0.05.

The remaining decedents (*n* = 95; 24%) had not received a diagnosis of cancer while alive; cancer was diagnosed in most after autopsies were performed (*n* = 90; Table 2). Of these 95 cases, 44 (46%) had never consulted a doctor, 32 (34%) had consulted a doctor without discontinuing medical care, and 17 (18%) had consulted a doctor at least once but discontinued medical care before death (Table 3). Of the 32 people who consulted a doctor without discontinuing medical care, cancer was not even suspected in 24. Cancer of the digestive system was the most frequent primary site (*n* = 16) among these decedents. Nine cases of abdominal symptoms (eg, abdominal pain, nausea) were noted among these 16 cases.

Mean age and sex did not significantly differ between diagnosed and undiagnosed cases (Table 2). The proportion of cases in which the colon was the primary cancer site was significantly larger among undiagnosed cancer cases; the proportion of gastric cancer was also higher among undiagnosed cases, but not significantly so (Table 2). The proportion of indigent patients was higher among undiagnosed cases (27%) than among diagnosed cases (16%; Table 2). The proportion of individuals who had received no medical consultations (*n* = 44) was higher among undiagnosed cases (Table 3), and indigent persons was the largest subgroup (*n* = 19; 43%) among those cases. The proportion of individuals who discontinued or received no medical consultation was larger among indigent persons than among non-indigent persons (Table 4).

**Table 4.** Medical consultation status, by economic status

	Indigent (*n* = 75)	Others (*n* = 325)	
Medical consultation	36 (48%)	243 (75%)	**
Discontinuation	18 (24%)	47 (14%)	*
No consultation	19 (25%)	25 (8%)	**
Unknown	2 (3%)	10 (3%)	

Values in parentheses indicate percentages of the total (*n* = 75; *n* = 325).

**P < 0.01, *P < 0.05.

## DISCUSSION

The results of this study suggest several ways to improve medical services and care provided to cancer patients in Tokyo, Japan. The similarity of the primary cancer site among the decedents and in the 2009 census report suggests that no particular type of cancer was responsible for sudden unexpected death.

Most of the decedents (*n* = 232, 58%) had received a diagnosis of advanced or terminal stage cancer during their lifetime and did not refuse medical consultation. In addition, most had symptoms of general weakness before death and went alone to a large hospital. However, they did not receive medical care at home, which suggests that too few physicians are experienced in terminal and palliative home care and can offer patient care when death is imminent. General practitioners (GPs) are key providers of palliative care for cancer patients in other countries,^11^ and palliative care is critical, as many patients with a terminal illness spend the final year of their life at home under the care of a GP and a primary health care team.^12^ However, a previous study showed that Japanese GPs have little experience in caring for terminally ill patients who choose to die at home.^13^ Thus, educating GPs about opioids and psychiatric medications,^13^ as well as implementing clinical pathways for pain management,^14^ could improve the quality of palliative care for cancer patients in Japan. In addition, close relationships between large hospitals and GPs are needed in order to supply adequate medical services to cancer patients.

We observed discontinuation of medical care or lack of consultation in 109 cases (27% of all cases; Table 3), and the proportion was higher among indigent persons (Table 4). A previous medicolegal study found that the proportion of cancer deaths was higher among homeless persons than among non-homeless individuals.^15^ The number of homeless people in Japan has been steadily decreasing,^16^ and many homeless people receive welfare payments and adequate housing. Establishing a system to perform medical checkups when individuals apply for welfare payments may be one of the best measures for early detection of cancer in this population. In addition, future studies should attempt to identify factors that contribute to interruption of cancer treatments among indigent persons, similar to previous surveys of tuberculosis.^17^

Gastric and colorectal cancers were more prevalent among undiagnosed cases (Table 2). The participation rate in nationwide gastric and colorectal cancer screenings was about 30% in 2010, which is considerably lower than in other developed countries.^18^ This highlights the need for measures that increase participation rates. In addition, physicians must carefully consider the possibility of cancer in elderly patients with digestive complaints.

This study has several limitations. The study sample did not include cases of natural death, as such deaths are not reviewed by medical examiners. We assumed that most such cases are individuals with a definite diagnosis of cancer who die in a hospital or hospice; thus, the proportion of patients who discontinued or did not receive medical care may be larger among this study sample than among cases of natural death. These differences might have led to the slightly lower age distribution in this study sample than in the census report, due to the insufficient medical care received by the former (Figure). Consequently, the results cannot necessarily be generalized to all cancer deaths.

In conclusion, there are many opportunities to improve medical services to cancer patients in the community. This analysis suggests that in Tokyo there is a shortage of doctors who are experienced in terminal and palliative care and who are able to ensure adequate patient care and follow-up. In addition to educating GPs on the care of terminal patients, the quality of medical services for cancer patients can be improved by expanding efforts to diagnose and monitor cancer patients, particularly among vulnerable populations such as welfare recipients, and by increasing participation rates for gastric and colorectal cancer screening.

## ONLINE ONLY MATERIALS

Abstract in Japanese.
