# Supplementary material for: Characteristics of Sudden Unexpected Cancer Deaths Investigated by Medical Examiners in Tokyo, Japan (2009)
Source: J Epidemiol. 2014 Mar 5;24(2):141–5. doi: 10.2188/jea.JE20130087 (PMC3956693; doi:10.2188/jea.JE20130087)
Supplement: Abstract in Japanese. [file je-24-141-s001.pdf]

東京都 23 区における悪性腫瘍による異状死の実態調査（2009 年）

鈴木秀人、谷藤隆信、阿部伸幸、福永龍繁
東京都監察医務院

（背景）監察医制度が施行されている東京都 23 区では悪性腫瘍による異状死が毎年約 400 件発生しているが、当該事例の生前診断等の疫学的事項の詳細は明らかにされていない。

（方法）2009 年に東京都監察医務院で取り扱った全異状死例から死因が悪性腫瘍である事例を抽出し（400 例）、生前の医療機関受診状況・診断、経済状況につき調査した。

（結果）大部分の事例（232 例、58%）は生前に終末期癌と診断されており、衰弱しているにも関わらずその多くは在宅医療を受けていなかった。一方、95 事例（24%）は生前未診断であったが、生前診断群と比較すると生活困窮者、医療機関未受診者、大腸癌の割合が有意に高かった。生活困窮者は医療機関未受診者の 43%を占め、医療機関受診状況に関して非生活困窮者と比較すると治療中断もしくは未受診の割合が有意に高かった。

（結論）悪性腫瘍に対する医療の質をさらに向上させるためには、開業医に対する終末期医療教育、生活困窮者に対する検診と医療の継続、大腸癌検診の受診率の向上が必要であると考えられる。

キーワード ; 悪性腫瘍、突然死、監察医、死因統計、終末期医療
